# RSM–GA Based Optimization of Bacterial PHA Production and *In Silico* Modulation of Citrate Synthase for Enhancing PHA Production

**DOI:** 10.3390/biom9120872

**Published:** 2019-12-12

**Authors:** Apoorva Rao, Shafiul Haque, Hesham A. El-Enshasy, Vineeta Singh, Bhartendu Nath Mishra

**Affiliations:** 1 Department of Biotechnology, Institute of Engineering and Technology, Dr. A.P.J. Abdul Kalam Technical University, Lucknow, Sitapur Road, Lucknow 226021, Uttar Pradesh, India; apoorva.jai18@gmail.com; 2 Research and Scientific Studies Unit, College of Nursing & Allied Health Sciences, Jazan University, Jazan 45142, Saudi Arabia; shafiul.haque@hotmail.com; 3 Institute of Bioproduct Development (IBD), Universiti Teknologi Malaysia (UTM), Skudai, Johor Bahru 81310, Malaysia; henshasy@ibd.utm.my; 4 School of Chemical Engineering, Faculty of Engineering, Universiti Teknologi Malaysia (UTM), Skudai, Johor Bahru 81310, Malaysia; 5 City of Scientific Research and Technological Applications, New Burg Al Arab 21934, Alexandria, Egypt

**Keywords:** polyhydroxyalkanoates, PHA, response surface methodology, molecular docking, optimization, genetic algorithm

## Abstract

The inexhaustible nature and biodegradability of bioplastics like polyhydroxyalkanoates (PHAs) make them suitable assets to replace synthetic plastics. The eventual fate of these eco-friendly and non-toxic bioplastics relies upon the endeavors towards satisfying cost and, in addition, execution necessity. In this study, we utilized and statistically optimized different food (kitchen-/agro-) waste as a sole carbon/nitrogen source for the production of PHA at a reduced cost, indicating a proficient waste administration procedure. Seven different types of kitchen-/agro-waste were used as unique carbon source and four different types of nitrogen source were used to study their impact on PHA production by *Bacillus subtilis* MTCC 144. Among four different studied production media, mineral salt medium (MSM) (biomass: 37.7 g/L; cell dry weight: 1.8 g/L; and PHA: 1.54 g/L) was found most suitable for PHA production. Further, carbon and nitrogen components of MSM were optimized using one-factor-at-a-time experiments, and found that watermelon rind (PHA = 12.97 g/L) and pulse peel (PHA = 13.5 g/L) were the most suitable carbon and nitrogen sources, respectively, in terms of PHA (78.60%) recovery. The concentrations of these factors (sources) were statistically optimized using response surface methodology coupled with the genetic algorithm approach. Additionally, in order to enhance microbial PHA production, the interaction of citrate synthase, a key enzyme in the TCA cycle, with different known inhibitors was studied using *in silico* molecular docking approach. The inhibition of citrate synthase induces the blockage of the tricarboxylic cycle (TCA), thereby increasing the concentration of acetyl-CoA that helps in enhanced PHA production. Molecular docking of citrate synthase with different inhibitors of PubChem database revealed that hesperidin (PubChem compound CID ID 10621), generally present in citrus fruits, is the most efficient inhibitor of the TCA cycle with the binding score of –11.4 and warrants experimental validation. Overall, this study provides an efficient food waste management approach by reducing the production cost and enhancing the production of PHA, thereby lessening our reliance on petroleum-based plastics.

## 1. Introduction

The discovery of synthetic plastic was a boon for societal development, but excessive and indiscriminate use of this material in every sector tremendously increased the amount of non-biodegradable waste material all over the world, and now it has become a curse for our environment. Due to its non-degradable nature, it is accumulating continuously in the environment and has put an adverse effect on all of our biological systems, including habitat [1]. All of these activities lead to imbalance of our ecosystem. Proper disposal of synthetic plastics is a major issue, as these are xenobiotic in nature and hence resistant to microbial degradation processes. Synthetic plastics persist in our ecosystem for several years as their molecular size is very big, which is the major reason for the resistance towards microbial degradation [2]. The widely used methods of getting rid of synthetic plastics are either incineration or recycling. Incineration generates a surplus amount of CO_2_, along with some highly toxic gases that are accountable for global warming and air pollution; whereas, the recycling process is extremely expensive [3].

Polyhydroxyalkanoates (PHAs) consist of a family of biodegradable, biocompatible polyesters with zero toxic waste, and are completely recyclable into organic waste. PHAs are synthesized by bacteria as an intracellular carbon and energy reserve material. PHAs are chemical biopolymers (bioplastic) of hydroxyalkanoic acids [4,5]. PHAs are lipid inclusions that are stored in bacterial cells in the form of granules (0.2–0.5 µm). The core of PHAs is enclosed by phospholipids [6]. The aggregation of PHAs takes place when bacteria undergo certain starvation conditions, such as when nitrogen is present in depleted amount or other substrates such as carbon source (electron donor) are present in excessive amount [7].

Although, in a recent report, Koller (2017) questioned the sustainability of biopolymers (PHAs) in comparison with synthetic plastics and stated that without envisaging the entire life cycle of bioplastics, it is difficult to conclude if they perform better in terms of environmental benefit than their synthetic counterparts [8]. However, he encouraged prolific and focused research in the entire PHA production chain that comprises finding novel producer strains, feedstock (media) selection, fermentation and process engineering, bioreactor designing, and downstream processing to achieve the criteria of sustainability [8].

The major obstacle in the commercialization of this bioplastic (i.e., PHAs) is its high production cost. Hence, various efforts have been made in the recent past to minimize the production cost of PHAs by using inexpensive, sustainable, and renewable carbon and nitrogen sources as a substrate for PHA production [9,10]. For example, the use of various food (kitchen-/agro-) waste materials (like fruit or vegetable peels) as a carbon source might be a promising option for cost-effective production of PHAs at commercial scale. In the recent past, plenty of attempts have been made to minimize the production cost by utilizing cheap and sustainable carbon sources as a substrate for PHA production [11,12,13]. The use of waste materials like organic product peels or vegetable peels as a carbon source is an exceptionally economic technique for industrial-scale production of PHAs.

Screening and optimization of fermentation medium are the major influential factors that play a critical role in the cell growth and expression of the preferred metabolite, hence affects the overall productivity. Earlier studies have reported that several conventional and statistical methods have been used extensively for medium optimization for metabolite production [14]. The conventional non-statistical one-factor-at-a-time (OFAT) approach is excessively time-consuming, labor-intensive and deficient in accurate finding of the critical factors that impact the desired metabolite’s production, and lacks in deciphering the interactions among the factors under investigation [15]. In order to prevail over these shortcomings, various statistical methods alone or in combination with artificial intelligence techniques have been used efficiently for the optimization of the medium constituents [14,16,17,18]. The response surface method (RSM), a statistical optimization technique, employs experimental factorial designs like central composite design (CCD) for optimizing any process output, and defines the behavior of the response in the selected design space [16,17]; wherein, CCD explores the interaction effect of the factors predominantly influencing the product formation. The experimental runs of the CCD work as inputs for RSM in finding the mathematical model that links process parameters (factors) and outcome. The mathematical model generated by RSM serves as a fitness function for a genetic algorithm (GA) to find out the optimum concentrations of the parameters involved in the process for maximum process output. A genetic algorithm is a heuristic search approach employed in artificial intelligence (AI) and computing, and is used for finding optimized solutions against search problems of constrained or unconstrained nature (for large and complex datasets) based on the theory of natural selection and evolutionary biology. GA randomly chooses the individuals from the present population to behave as parents, and exploits them for producing offspring for the next generation on the basis of the rules of selection, crossover, and mutation. Over successive generations, the population “mutates” in the direction of an optimal solution [18,19].

Keeping aforementioned facts in view, the present study aimed to reduce the production cost of PHAs by employing various kitchen-/agro-waste materials, like fruit or vegetable peels, as a sole carbon source, followed by RSM coupled GA-based optimization of the parameters for enhanced production of PHAs. In addition, in silico molecular docking studies were performed for elucidating the interaction of citrate synthase, a key enzyme of the tricarboxylic cycle (TCA) or Krebs cycle with different known inhibitors (51 compounds available in PubChem database) to enhance the production of PHAs; as the inhibition of citrate synthase increases the concentration of acetyl-CoA that possibly assists in enhanced PHA production. The addition of potent citrate synthase inhibitor(s) after experimental validation in the bacterial growth media, along with optimization of process parameters using the RSM–GA amalgamated approach for enhanced PHA production using cheaper carbon/nitrogen (C/N) sources, might prove an economically feasible way of replacing hazardous synthetic plastics. 

## 2. Materials and Methods

### 2.1. Microorganism and Fermentation Condition

*Bacillus subtilis* MTCC 441 was used for the production of bioplastic (PHAs) in mineral salt medium (MSM) [composition (g/L): Urea (1.0), yeast extract (0.16), KH_2_PO_4_ (1.52), Na_2_HPO_4_ (4.0), MgSO_4_∙7H_2_O (0.52), CaCl_2_ (0.02), Glucose, and trace element solution contained (g/L): ZnSO_4_∙7H_2_O (0.13), FeSO_4_∙7H_2_O (0.02), (NH_4_)6MO7O_24_. 4H_2_O (0.06), and H_3_BO_3_ (0.06)]. All media components were of analytical grade and solvents were purchased from HiMedia Laboratories (India).

Various disposed of kitchen-/agro-waste peels were collected from local vegetable/fruit market areas of Lucknow, Uttar Pradesh, India. The collected waste peels were washed thoroughly with water to remove dust/soil particles, chopped into small pieces, and dried completely in a hot air oven at 60 °C. Fully dried vegetable/fruit peels were grounded as powder using a mortar and pestle, and further used as a substrate for the production of PHAs.

PHA production was performed in the fermentation medium using *B. subtilis* MTCC 144 under biphasic growth conditions as per the protocol given by Chee et al. with minor modifications [11]. Briefly, the production medium was autoclaved at 121 °C for 15 min and inoculated with 3% inoculums (24-hour-old culture of *B. subtilis*). Fermentation was carried at 150 rpm, 37 °C for 48 h. The culture was centrifuged at 10,000 rpm for 20 min under sterilized condition. The cell pellet obtained from the first-stage culture was inoculated in nitrogen-deficient medium of second phase and incubated at 150 rpm for 48 h at 37 °C. After extraction, the results were compared with single phasic production. All of the experiments performed in the study were done under biphasic growth conditions at shake flask level in triplicate.

### 2.2. Selection of Production Medium

Four different media [nutrient broth (NB) (g/L): Peptone 5.0, sodium chloride 5.0, beef extract 1.5, yeast extract 1.5; Luria Bertani (LB) (g/L): Casein enzyme hydrolysate 10; yeast extract 5; sodium chloride 10; M6-Medium (M6) (g/L): Glucose 20, beef extract 5, (NH_4_)_2_SO_4_ 1.0, KH_2_PO_4_ 0.6 and MgSO_4_ 1.0, and mineral salt medium (MSM; composition given in the above section)] were prepared. Media, glucose, and trace elements’ stock solution were autoclaved separately at 15 lbs pressure (121 °C) for 15 min. Glucose and 1% trace elements’ stock solutions were added and inoculated with 24-hour-old inoculum of *B. subtilis,* and incubated at 37 °C at 150 rpm for 48 h, followed by centrifugation at 10,000 rpm for 20 min under sterilized condition. The cell biomass was lyophilized and dried completely. PHAs were then extracted from the dried cell pellet by using the solvent extraction method.

### 2.3. Extraction and Quantification of PHA

The cell pellets obtained from the second stage culture was homogenized and then dried. The extraction of PHAs was tried using three different methods, i.e., methanolic method, dispersion of NaClO and CHCl_3_, and sodium hypochlorite method. However, based upon PHA content recovered, finally the extraction was done by sodium hypochlorite method [20]. Briefly, the powdered biomass was treated with sodium hypochlorite, followed by stirring at 37 °C for 10 min. The material was centrifuged at 10,000 rpm for 20 min and the layer of sodium hypochlorite was discarded. The cell pellets were washed with the solvent mixture containing equal ratios of diethyl ether, methanol, and acetone. The washed pellets were treated with boiling chloroform and the solvent was evaporated under reduced pressure. PHA granules obtained after evaporation were dissolved in boiling chloroform and air-dried to obtain PHA powder. The quantification of extracted PHAs was done using crotonic acid assay using commercial PHAs (Sigma-Aldrich, USA) as a reference standard [21,22].

### 2.4. Selection of Carbon and Nitrogen Source

The effect of various C/N sources on the synthesis of PHAs by *Bacillus subtilis* was evaluated by separately incorporating seven different types of kitchen waste peels as a unique carbon source, and four different types of nitrogen source 4% (*w*/*v*) in MSM medium during first-stage culture. For this, replacement experiments were performed to identify the carbon and nitrogen sources, cheaper and better than glucose or urea [18]. Firstly, for carbon source, the powdered wastes (fruit waste, vegetable waste, green pea peels, orange peels, papaya peels, musk melon peels, and watermelon rinds) materials were added as carbon source in the place of glucose in MSM medium. PHA production was carried out in MSM media (devoid of glucose) using *B. subtilis* MTCC 144 under biphasic growth conditions. Briefly, the modified MSM media (food waste material as a carbon source) and trace element solution were autoclaved separately at 121 °C for 15 min. Afterwards, both the solutions were mixed and inoculated with *B. subtilis* culture and incubated at 150 rpm for 48 h at 37 °C. Following the incubation, the culture was centrifuged at 10,000 rpm for 20 min under sterilized condition and the cell pellet was used to inoculate the nitrogen-deficient medium of second phase and further incubated at 150 rpm for 48 h at 37 °C.

Likewise, after selection of the best carbon source from the above-mentioned experiments using various food (kitchen-/agro-) waste, further replacement experiments were performed for the selection of other nitrogen sources instead of urea and yeast extract as medium components. The culture was then centrifuged at 10,000 rpm for 20 min. The weight of the biomass was measured. This biomass was further used as an inoculum for the second-stage culture under nitrogen-deficient condition. Following the incubation period, the culture was harvested and PHAs were extracted as mentioned above. Cell dry weight and PHA content were measured to analyze the overall product recovery.

### 2.5. RSM-Based Optimization of PHA Production

Response surface methodology was used to describe the effect of independent variables, alone or in combination on the process. Two factors (concentrations of carbon and nitrogen) that significantly affect PHA production were optimized by using RSM 5 level 2 factorial design. A CCD of 10 experiments (5 levels of each factor) was conducted to describe the relationship between the independent variables and PHA content (dependent variable). Statistica V. 10.0 was used for the regression and graphical analysis of the results obtained from CCD. A second-order polynomial response equation (of the form given below) comprising linear, quadratic, and interaction terms was obtained.

Y=b^º^+∑ b_i_X_i_+∑b_i_^2^X_i_^2^+∑b_ij_X_i_X_j_ (1)

where Y is weight of biomass in grams, b^º^ is the intercept, b_i_ is the coefficient for linear effect, b_i_^2^ is the coefficient for quadratic effect and is responsible for curvatures in the model, and b_ij_ is the coefficient for interaction effect.

### 2.6. GA Optimization

The second-order polynomial model obtained from the application of RSM was further subjected to GA program of MATLAB suite in order to get the optimum concentration of the dependent variable for optimum production of PHA. The input parameters considered in “ga” function were population type as “double vector”; pop init range as [2 × 1 double]; population size as 200; elite count as 2; crossover fraction as 1; migration direction as “forward”; migration interval as 20; migration fraction as 0.2000; generations as 100; time limit as Inf; fitness limit as −Inf; stall gen limit as 50; stall time limit as 20; initial population and scores as []; plot interval as 1; creation fcn, fitness scaling fcn, selection fcn and crossover Fcn as @gacreationuniform, @fitscalingrank, @selectionstochunif and @crossoverscattered, respectively; mutation fcn as {[1x1 function_handle] [1] [1]}; hybrid fcn as []; display as “off”; plot fcns as {[1x1 function_handle] [1x1 function_handle]}; output fcns as []; vectorized as “off”.

### 2.7. Characterization of PHAs

#### 2.7.1. (i) FTIR Analysis of PHAs

The dried sample of PHAs extracted by sodium hypochlorite method was subjected to Fourier transform infrared (FTIR) spectroscopy study. The analysis was performed by using KBr Pellet method [23], and the absorption was recorded in the range of 4000–450 cm^−1^ (Perkin-Elmer Spectrum-II spectrometer, MA, USA).

#### 2.7.2. Thin Layer Chromatography of PHAs

In order to perform thin layer chromatography (TLC), the extracted PHA was dissolved in chloroform, and chromatography was performed on a TLC plate coated with silica (230–400 mesh size) [24]. Methanol and chloroform were used as solvent system in various ratios. After completion of the TLC run, the plates were air dried and visualized under iodine chamber and UV chamber; afterwards, the R_f_ values were calculated.

#### 2.7.3. ^1^H-NMR

^1^H-NMR spectrum of the sample (i.e., PHAs) was obtained by using a Bruker AvIII HD-300 spectrophotometer. The spectrum was recorded at 300 MHz against TMS (tetramethylsilane) as internal reference standard. Approximately 5 mg of PHAs was dissolved in 2 mL CDCl_3_ (deuterated chloroform) for sample preparation.

#### 2.7.4. X-Ray Diffraction (XRD) Analysis

The crystalline nature of the produced PHAs was determined by using X-ray diffractogram with K-β filter (40 mA, 40 KV) source of radiation done by powder method. The sample of PHAs was freeze-dried in a capillary tube. The scan speed was kept as 3.000 degree/min and the scan range was in between 10,000–80,000 degrees.

### 2.8. In Silico Modulation of Citrate Synthase for the Enhancement of PHA Production

#### 2.8.1. Target Selection and Preparation

The inhibition of citrate synthase induces enhanced accumulation of acetyl-CoA, thereby contributing to increased PHA synthesis. The significant involvement of citrate synthase enzyme in PHA production has been exploited as a potential target (protein details available at www.rcsb.org) for maximizing PHA production using in silico approach. The Protein Data Bank (PDB) is a depository for three-dimensional (3D) structure database of large biological molecules, especially proteins. This database comprises 3D structures of proteins established from X-ray crystallography or NMR spectroscopy data. The structural details of the target protein (i.e., citrate synthase) were retrieved from the PDB database and downloaded in PDB format.

#### 2.8.2. Library Preparation of Ligands

The library of the ligand (51 PubChem compounds) was prepared according the literature search and the structural details of the compounds were downloaded in SDF format from the PubChem Database (http://pubchem.ncbi.nlm.nih.gov). The format of the downloaded files was converted into PDB by using the Open Babble software program. The details of the compounds used as inhibitors, such as PubChem CID, chemical name, molecular formula, and molecular weight, were also retrieved from the PubChem database (kindly refer to Supplementary Information Table S1).

#### 2.8.3. Molecular Docking

The docking studies of PubChem compounds with citrate synthase were performed using molecular docking program Autodock Vina 4.2 (www.autodock.scripps.edu/). Docking combines energy estimation through pre-calculated grids of attraction potential, engaging various search algorithms to find the best binding positions for a definite ligand on a target protein [25]. In the case of attachment of any ligand with citrate synthase protein structure, it was removed by using Discovery Studio suite for performing molecular docking. For effective and accurate docking, polar hydrogen atoms and Kollman charges were added to the protein structure, and water molecules were removed. This was followed by saving the protein into PDBQT format and the selection of 3D (X,Y,Z) grid box using DoGsite scorer. For ligand preparation, the molecule was opened in ligand tab, followed by the selection of torsion tree and torsion count wizard showing bonding properties. During the preparation, all of the active bonds were made non-rotatable and saved in PDBQT file format. Afterwards, the command prompt was opened and the address of the vina.exe and protein, ligand file in PDBQT file format was mentioned. After processing, the results of docking were obtained.

## 3. Results and Discussion

**Figure 1:**
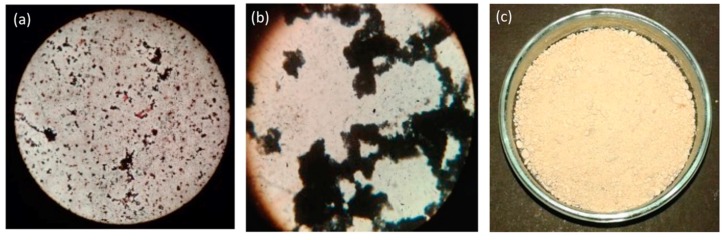
Production. (**a**) First phase: Biomass production stage. (**b**) Second phase: PHA aggregation stage. (**c**) Third phase: PHAs after sodium hypochlorite extraction.

*Bacillus subtilis* is a well-known PHA producer [26]; here in this study, *B. subtilis* MTCC 144 was exploited for PHA production considering two phase production schemes during the experiments. Before moving ahead for the experimental part, the presence of PHAs was verified with Sudan black dye staining protocol [27], which is generally used as a preliminary screening agent for lipophilic compounds. Earlier, Bhuwal et al. successfully used Sudan black dye for the screening of PHA-producing bacteria [28]. In the present study, the used strain *B. subtilis* MTCC 144 also gave positive results in the staining experiment (Figure 1a,b). After confirming the presence of PHA, the aggregated PHAs were extracted via sodium hypochlorite method (Figure 1c).

### 3.1. Effect of Various Media on PHA Production

**Figure 2:**
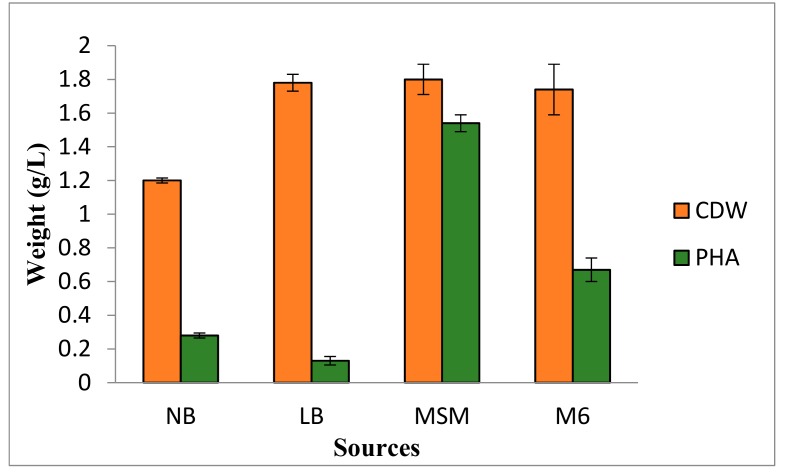
Microbial dry cell weight and PHA content in four tested production media. NB, Nutrient broth; LB, Luria Bertani; MSM, mineral salt medium; M6, YMG medium.

In the medium, carbon and nitrogen contents, which are utilized by the bacteria, decide the productivity of PHAs. Therefore, to enhance the PHA production, the effect of various media on PHA production was studied, and the results are summarized in Figure 2. It is evident from the results that the maximum cell dry weight and PHA production were found in MSM medium (biomass: 37.7 g/L; cell dry weight: 1.8 g/L; and PHA: 1.54 g/L) compared to Luria Bertani, nutrient broth, and YMG (M6). PHA production in terms of PHA concentration, i.e., grams of biopolymer per liter of the cultivation medium, was found minimum in the case of Luria Bertani medium (0.13 g/L).

### 3.2. Effect of Carbon and Nitrogen Sources Derived from Various Kitchen-/Agro-Waste on PHA Production

PHA accumulation is favored by sufficient availability of a suitable carbon source suffice with finite supply of macro-components (phosphorus, nitrogen, and dissolved oxygen) and micro-components (magnesium, sulphate, iron, potassium, manganese, copper, sodium cobalt, tin, and calcium) [2]. Various kitchen-/agro-wastes have been reported as unique carbon sources in the past [3,29]. Therefore, different kitchen-/agro-wastes were taken into consideration as carbon and nitrogen sources to examine their effect on PHA content (Table 1).

Among the selected carbon and nitrogen sources, the maximum conversion was observed in the case of green pea, musk melon, water melon, and papaya peels (nearly 78%), whereas the minimum conversion was observed for orange peel (55.07%). Our findings are in line with the previous study of Kumar et al., in which they reported 61% PHA production by *Bacillus cereus* using potato starch as a sole carbon source [3]. Likewise, Kulkarni et al. found that PHA production efficiency of *Halomonas campisalis* by using banana peels as a unique carbon source was 22% [29]. In another study, Gomaa reported 48% PHA production by *Bacillus subtilis* using cane molasses as a unique carbon source [26]. In the present experimental study, mixed vegetable peels (63.27%), green pea shells (77.89%), muskmelon rind (78.56%), watermelon rind (78.61%), and papaya peels (77.67%) resulted better yields than previous findings. The excess of carbon source present in the medium produced during the first stage of the growth allowed the microbes to attain the maximum growth. The cells were harvested after 48 h and used to inoculate the nitrogen-deficient conditions, leading to physiological alteration of the bacterial cells that facilitated PHA production. The percentage conversion of PHA production by using different nitrogen sources were as follows: Peptone 78.61%, pulse peels 69.20%, beef extract 61.01%, and yeast extract 78.62%. A total amount of 2.4% PHA was obtained using yeast extract as a nitrogen source by *B. subtilis* MTCC 144. However, the earlier study of Shah reported 7.14% PHB recovery using peptone as a nitrogen source by *B. subtilis* [30].

### 3.3. Statistical Optimization

Central composite design of RSM considering two factors was used to optimize carbon (watermelon peel) and nitrogen (pulse peel) concentration in the medium to obtain maximum PHA concentration. The results of statistical CCD optimization for maximizing PHA production are summarized in Table 2.

The efficiency of the generated model was determined with the help of analysis of variance (ANOVA), which was evaluated by Fisher’s statistical analysis. The developed model has a correlation coefficient (R) = 98.03% (Table 3), which indicates that the interaction between the variables is significant, and a determination coefficient (R^2^) = 96.10%, which suggests that the generated model is significant and is capable of interpreting 96.10% of the input data [17].

Further, the regression coefficient analysis (Table 4) suggests that the linear and square effect of the carbon source is relatively more effective than the nitrogen source for enhanced PHA production.

The model generated through software is expressed in the form of a polynomial equation. By solving the equation, the optimum concentration of all (two) variables (in the considered range of concentration) having optimum biomass weight for the production of PHAs can be attained.

**Figure 3:**
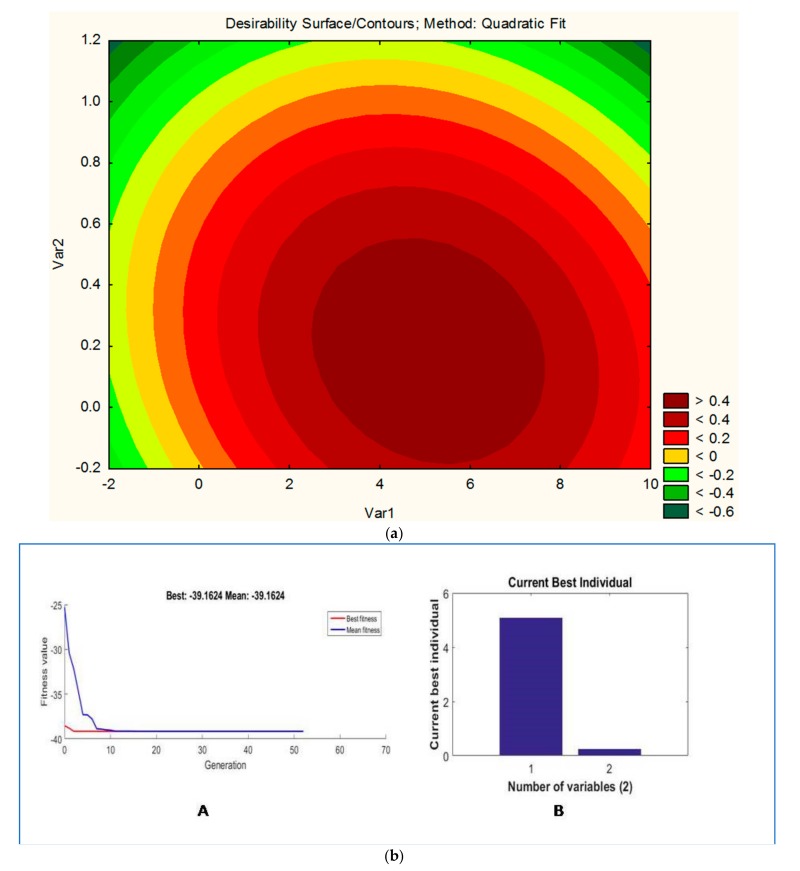
(**a**) Contour plot showing the effect of Var1 (watermelon rind) and Var2 (pulse peel) on desirability. Note: Desirability is the response, i.e., weight of biomass for the production of PHAs. (**b**) Genetic algorithm analysis showing generations until the optimum PHA level is obtained. (**A**) Graph between current generation vs. fitness values; (**B**) graph between current best individual vs. number of variables.

Y = −0.4595 + 13.053 × Var1 − 1.320 × Var1^2^ +44.166 × Var2 − 104.756 × Var2^2^ + 1.546 × Var1 × Var2 (2)

where Y is the response, i.e., weight of biomass (gm) of PHA produced, and Var1 (watermelon rind) Var2 (pulse peel) are the coded values of the test variables 1 and 2. Response contour plot (Figure 3a) is helpful in understanding the effect of individual variables.

The graph was plotted between carbon concentration (Var1) and nitrogen concentration (Var2). The graph suggests that a higher value of Var1 (carbon concentration) and a lower value of Var2 (nitrogen concentration) are required to maximize the weight of biomass for the production of PHAs (Figure 3a).

### 3.4. GA-Based Optimization

The application of GA to optimize the RSM model is a well-established technique and used in diverse studies [14,31]. In the present study, the application of GA predicts that if carbon and nitrogen sources will be taken 5.09 and 0.243 g/100 mL, respectively, the maximum PHA content will be 39.16 g/100 mL. This is nearly 188% higher than the yield from the unoptimized medium (13.5 g/L). This prediction was further validated experimentally and the yield obtained was 38.5 g/L, which was very close to the predicted one.

### 3.5. PHA Characterization

**Figure 4:**
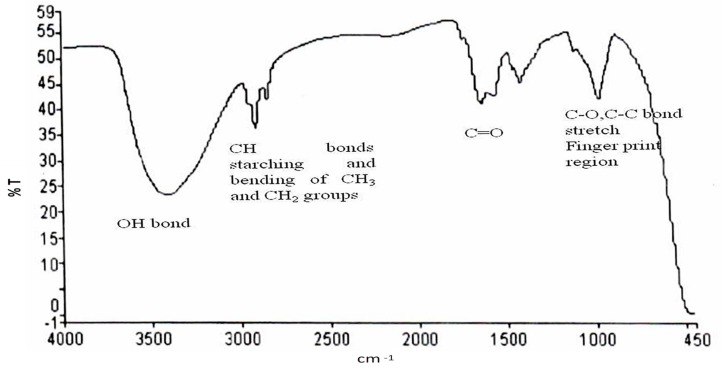
FTIR spectra of the extracted PHA.

The polymer PHA produced by using watermelon rind as a carbon source was further investigated for the identification of chemical functional group(s). The depiction of functional groups through FTIR spectroscopy predicts the structure of the produced PHAs (Figure 4).

The sample showed the strongest band at 3414.09 cm^−1^, corresponding to the hydroxyl group, and other vibrational peaks were found at 2920.9 cm^−1^ which corresponds to CH bond of CH_3_ and CH_2_ groups, 1638.90 cm^−1^ which corresponds to C=O, 1414.56 cm^−1^ which corresponds to CH_3_, 972.25 cm^−1^ which corresponds to C–O, C–C bond stretch and indicates the presence of PHAs [32]. Earlier, Nair et al. reported that the band obtained at 1636 cm^−1^ shows the characteristic feature of the C=O group, and the band obtained at 1726.13 cm^−1^ reflects the presence of C=O (amide group) [33]. Likewise, in the recent past, Vega et al. reported that the band in the range of 3200–3500 cm^−1^ shows OH group and the band at 2928 cm^−1^ is the characteristic feature of CH vibrations for PHAs [34].

During TLC analysis, PHAs in the form of polyhydroxybutyrate (PHB) appeared as yellowish–green spots on the TLC plate. The R_f_ value obtained was 0.875, which was higher than the normal R*_f_* value. The R*_f_* value of PHA tends to increase during the polymerization (whereas the R*_f_* value decreases due to propanolysis, but this is not the case here as the sample was not propanolysed), hence the obtained R*_f_* value was a little high [24]. The extracted PHAs (in the form of PHB) was quantified by Crotonic acid assay, in which the chemical breakdown of PHB polymer occurs and results in the formation of monomer units [35]. During this assay, PHB treatment with concentrated sulphuric acid caused transformation of monomer units of hydroxyalkanoaic acid into crotonic acid, which absorbs UV radiations at 235 nm and aids in the estimation of relative amounts of PHB present in the sample [35]. The concentration of unknown PHB was estimated from the standard curve of PHB; the equation obtained from the standard curve was used for the quantification of PHAs (as PHB), and it was found to be 0.58 µg/L.

The peaks obtained during NMR studies revealed the existence of protons at chemical shifts 1.0, 1.75, 2.507, and 4.490 ppm. The signals at 1.0 and 1.75 ppm were characterized by methyl group, whereas the signals at 2.507 and 4.490 ppm were characterized by methylene and methine groups, respectively (data/figure not shown). The results obtained were congruent with earlier reports of Pillai et al. [36]. Both FTIR and NMR spectra of the extracted PHAs, along with crotonic acid assay, confirmed PHB form of the bioplastic production.

**Figure 5:**
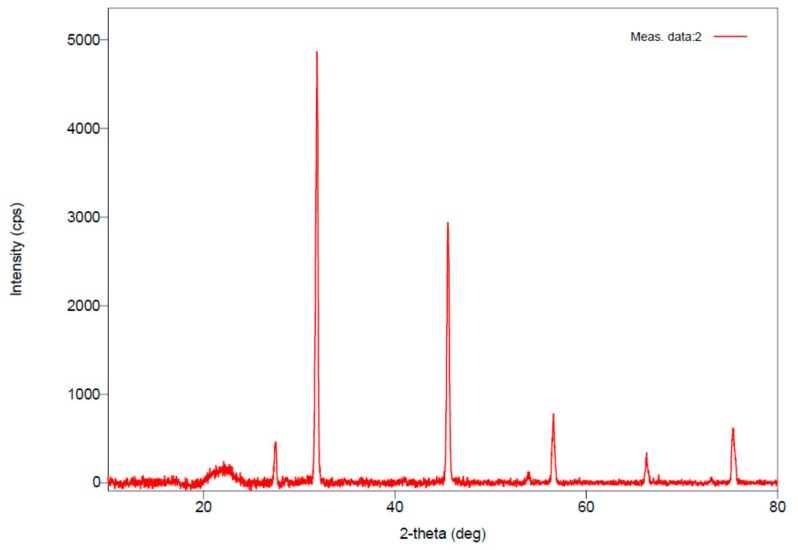
X-Ray Diffraction spectrum of the extracted PHAs.

In a recent study, Sedlacek et al. reported that microbes store PHA polymers in amorphous form, which has more elasticity and flexibility in comparison with crystalline [37]. During the extraction procedure (of PHAs), the amorphous form converts into crystalline form. The nature of the extracted PHAs, i.e., amorphous or crystalline, was confirmed through XRD study. The XRD pattern of the extracted PHAs is shown in Figure 5. Diffractogram of the obtained PHAs exhibited six prominent peaks at 27.512°, 31.859°, 45.535°, 56.501°, 66.319°, and 75.310°. These angles coincide with the earlier studies of Alashwal et al. [38] and Mohapatra [39]. The increased intensity of the peaks indicates that extracted PHAs have well organized packed crystalline structure [33]. The d-spacing of the analyzed sample of PHAs indicates that the sample has a well-arranged crystalline structure [38].

### 3.6. Molecular Docking Studies of Citrate Synthase

**Figure 6:**
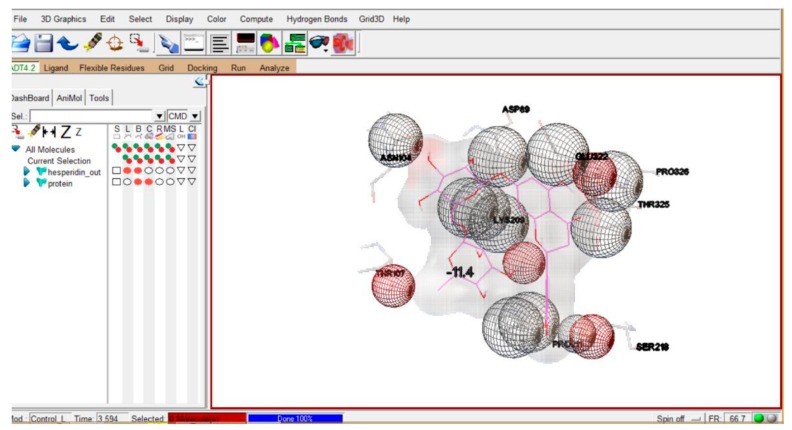
Protein (citrate synthase)–ligand (PubChem: CID ID 10621) interaction.

In the target protein citrate synthase, some possible sites—called binding sites—are present, where the ligand molecule may bind specifically. A molecular docking strategy includes a computational procedure of looking for an affirmation of the ligand that can fit both geometrically and chemically into the binding site of a protein. Docking calculations were used to foresee the binding pattern of the ligand molecule. The binding energy computations were expected to recognize the best drug candidate(s). The distinctive factors associated with ligand–protein interaction are electrostatic, electrodynamics, steric powers, and dissolvable-related powers (kindly refer to Supplementary Information Table S1). The most efficient binding score obtained was −11.4 (Figure 6).

Out of 51 tested PubChem compounds, the compound CID ID 10621 showed the most efficient binding energy of −11.4 (Figure 6). This interaction reflects that ligand CID ID 10621 binds very efficiently with the target protein (citrate synthase). The compound with CID ID 10621 is “hesperidin”, which is a type of flavanon glycoside and generally present in citrus fruits. In general, citrus peels contain hesperidin content in a greater concentration than other parts of the fruit, and this bioflavanoid has a variety of applications [40]. Hence, based upon the results of the above docking interaction, we can speculate that intervention of a potential inhibitor (ligand) helps in the modulation of the TCA biochemical cycle, and ultimately promotes the stress condition of a higher concentration of acetyl-CoA. As we know, acetyl-CoA and oxaloacetate combine with isocitrate with the help of citrate synthase to contribute in the TCA cycle; so, conjecture can be made that the binding of ligand compound CID ID 10621 with citrate synthase tends to the blockage of the TCA cycle and leads to greater accumulation/concentration of acetyl-CoA capable of efficiently participating in the PHA cycle, ultimately resulting in enhanced production of PHA. The inclusion of potent inhibitor (ligand) of citrate synthase (target protein), especially from natural sources (like citrus fruit peels, etc.), after experimental substantiation in the bacterial growth media coupled with optimization of process parameters using cheaper C/N sources [from food (kitchen-/agro-) waste] for enhanced PHA production, might provide an economical approach for minimizing environmental hazard due to synthetic plastics.

## 4. Conclusions

As the world’s population is increasing at an alarming rate, the consumption of conventional plastic is also increasing, which is the major cause of environmental pollution. Petroleum-based synthetic plastics do not degrade, resultantly accumulating in our environment for many years with uninterrupted release of toxins. The accumulation of these microplastics in our ocean ecosystem is a critical challenge. Since, petroleum resources are depleting, there is an urgent need of an alternative of these petroleum-based synthetic plastics, and PHAs are an exceptional substitute over synthetic plastics as they are biodegradable, non-toxic, and sustainable. Researchers across the globe are proposing various approaches for enhancing the production of PHAs in bacterial strains by various means, such as genetic engineering, recombinant DNA technology, etc. In the present study, we utilized different kitchen-/agro-waste as a sole C/N source following RSM coupled GA optimization of the PHA production, which gives an idea of a proficient waste administration strategy, along with cheaper PHA production. Among all of the utilized waste, watermelon peels (C source) and pulse peel (N source) showed ~78% PHA recovery. In silico docking studies were performed to study and determine future exploitation of the interaction of citrate synthase of the TCA cycle with known PubChem compounds to enhance the production of PHAs. It was found that compound CID ID 10621 (hesperidin), generally present in citrus fruits, is the most efficient inhibitor of the TCA cycle, with a binding score of −11.4, which suggests it use for experimental substantiation.

## Figures and Tables

**Table 1:** Effect of carbon and nitrogen sources derived from different kitchen-/agro-waste on PHA content.

S.No.		Cell Dry Weight (g/L)	PHAs (g)	% Conversion of PHAs
	**Carbon Source**			
1	Mixed fruit peels	8.5	4.9	57.64
2	Mixed vegetable peels	9.8	6.2	63.27
3	Green pea shells	11.26	8.77	77.89
4	Muskmelon peels	9.98	7.84	78.56
5	Watermelon rind	16.5	12.97	78.61
6	Papaya peels	15.0	11.65	77.67
7	Orange peels	19.39	9.68	49.93
	**Nitrogen Source**			
1	Peptone	16.5	12.97	78.61
2	Pulse peel	19.51	13.5	69.20
3	Beef extract	18.85	11.5	61.01
4	Yeast extract	12.02	9.45	78.62

**Table 2:** Central composite design (CCD) for the production of PHAs.

Runs	Carbon Concentration (g/100 mL)	Nitrogen Concentration (g/100 mL)	PHA Content (g)		
			Observed	Predicted	Residual
1	2	0.1	27.221	23.095	4.125
2	2	0.3	27.928	25.881	2.046
3	6	0.1	37.996	35.613	2.382
4	6	0.3	36.052	35.748	0.303
5	4	0.2	33.124	35.966	−2.842
6	0	0.2	2.25	4.228	−1.978
7	8	0.2	26.377	26.612	−0.235
8	4	0	29.38	31.526	−2.146
9	4	0.4	34.38	34.447	−0.067
10	4	0.2	34.38	35.966	−1.586

**Table 3:** Analysis of variance (ANOVA) for the quadratic model.

Source	SS	df	MS	F-value	Prob (*p*)
Whole model	896.0238	5	179.2048	19.72049	0.006392
Residual	36.34894	4	9.087235		

Note: High F- and low *p*-value suggests the significance of the model; SS: sum of squares, df: degree of freedom, MS: mean square; p: probability value

**Table 4:** Regression coefficient analysis.

Effect	Var3 Param.	Var3 Std. Err	Var3 *t*	Var3 *p*
Intercept	−2.8622	8.43462	−0.33934	0.751425
Var1	13.7336	2.43016	5.65131	0.004830
Var1^2	−1.2841	0.20846	−6.15993	0.003525
Var2	50.3486	48.60319	1.03591	0.358759
Var2^2	−74.4777	83.38407	−0.89319	0.422238
Var1*Var2	−3.3137	8.49142	−0.39025	0.716244

Var1: Carbon (watermelon peel); Var2: Nitrogen (pulse peel); Var3: PHA production.

## Supplementary Materials

The following are available online at https://www.mdpi.com/2218-273X/9/12/872/s1, Table S1: List of PubChem compounds used as inhibitors in the present study.
