# Supplementary material for: RSM–GA Based Optimization of Bacterial PHA Production and In Silico Modulation of Citrate Synthase for Enhancing PHA Production
Source: Biomolecules. 2019 Dec 12;9(12):872. doi: 10.3390/biom9120872 (PMC6995514; doi:10.3390/biom9120872)
Supplement: Supplementary file 1 [file biomolecules-09-00872-s001.pdf]

Supplementary Information

Table SI1. List of PubChem compounds used as inhibitors in the present study

S. No.	CID ID	IUPAC NAME	STRUCTURE	MOLECULAR WEIGHT	BINDING SCORE
1.	5284344	(E)-1-[(2S)-2-amino-2-carboxyethoxy]-2-diazonioethenolate	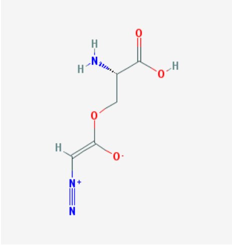	173.128 g/mol	-5.6
2.	2603	2-[(3-chlorophenyl)hydrazinylidene]propanedinitrile	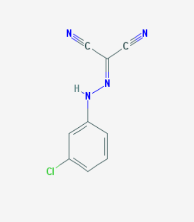	204.617 g/mol	-5.9
3.	10868	N,N'-dicyclohexylmethanediimine	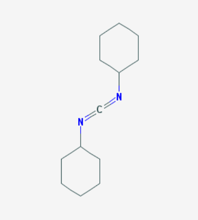	206.333 g/mol	-5.7

4.	3120	3-(3,4-dichlorophenyl)-1,1-dimethylurea	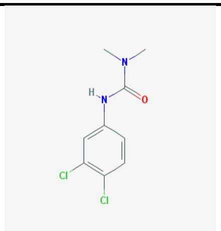	233.092 g/mol	-5.3
5.	6137	L-methionine	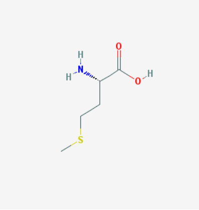	149.208 g/mol	-4.9
6.	6306	L-isoleucine	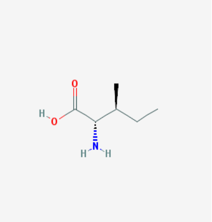	131.175 g/mol	-5.0
7.	12969119<br>2	2-fluoroacetic acid;hydrate	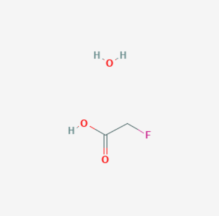	96.057 g/mol	-4.0
8.	784	hydrogen peroxide	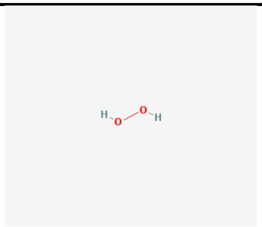	34.014 g/mol	-2.9

9.	10917	(3R)-3-hydroxy-4-(trimethylazaniumyl)butanoate	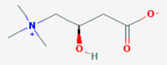	161.201 g/mol	-4.9
10.	586	2-[carbamimidoyl(methyl)amino]acetic acid	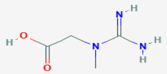	131.135 g/mol	-5.4
11.	4649	4-amino-2-hydroxybenzoic acid	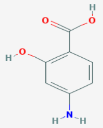	153.137 g/mol	-5.9
12.	222289	2,3-dibromo-3-phenylprop-2-enoic acid	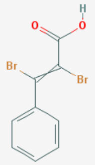	305.953 g/mol	-7.4
13.	6224	trisodium;2-hydroxypropane-1,2,3-tricarboxylate	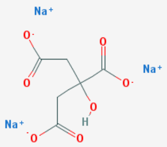	258.068 g/mol	-6.9

14.	60960	(2R)-2-amino-3-sulfanylpropanoic acid;hydrochloride	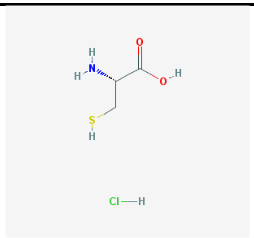	157.612 g/mol	-4.4
15.	6322	L-arginine	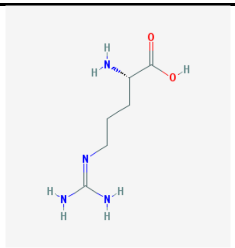	174.204 g/mol	-5.8
16.	5781	1,3-diazinane-2,4,5,6-tetrone	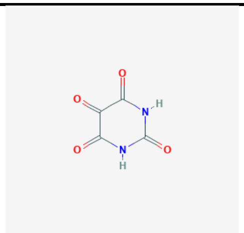	142.07 g/mol	-5.5
17.	1130	2-[3-[(4-amino-2-methylpyrimidin-5-yl)methyl]-4-methyl-1,3-thiazol-3-ium-5-yl]ethanol	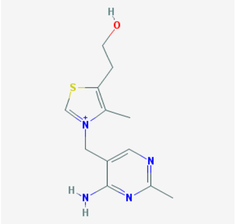	265.355 g/mol	-6.5

18.	493570	7,8-dimethyl-10-[(2S,3S,4R)-2,3,4,5-tetrahydroxypentyl]benzo[g]pteridine-2,4-dione	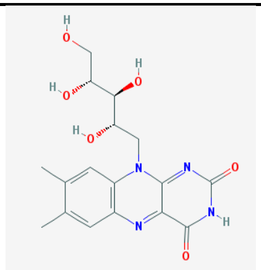	376.369 g/mol	-9.2
19.	938	pyridine-3-carboxylic acid	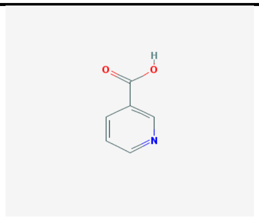	123.111 g/mol	-5.0
20.	864	5-(dithiolan-3-yl)pentanoic acid	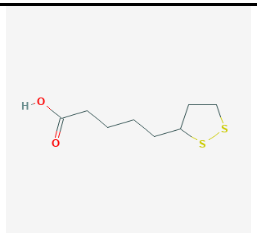	206.318 g/mol	-5.5
21.	6613	3-[[(2R)-2,4-dihydroxy-3,3-dimethylbutanoyl]amino]propanoic acid	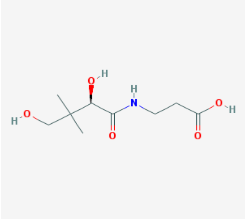	219.237 g/mol	-6.0

22.	445354	(2E,4E,6E,8E)-3,7-dimethyl-9-(2,6,6-trimethylcyclohexen-1-yl)nona-2,4,6,8-tetraen-1-ol	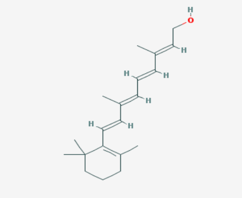	286.459 g/mol	-6.7
23.	14985	(2R)-2,5,7,8-tetramethyl-2-[(4R,8R)-4,8,12-trimethyltridecyl]-3,4-dihydrochromen-6-ol	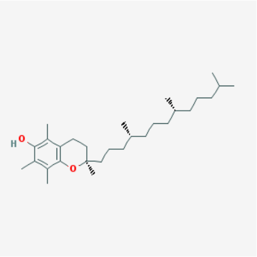	430.717 g/mol	-7.9
24.	54670067	(2R)-2-[(1S)-1,2-dihydroxyethyl]-3,4-dihydroxy-2H-furan-5-one	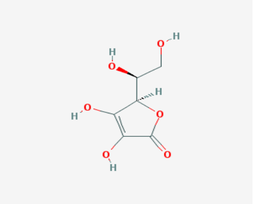	176.124 g/mol	-6.4
25.	31703	(7S,9S)-7-[(2R,4S,5S,6S)-4-amino-5-hydroxy-6-methyloxan-2-yl]oxy-6,9,11-trihydroxy-9-(2-hydroxyacetyl)-4-methoxy-8,10-dihydro-7H-tetracene-5,12-dione	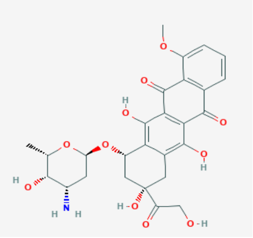	543.525 g/mol	-9.6

26.	439501	3-[(1R,3S,5S,8R,9S,10R,11R,13R,14S,17R)-1,5,11,14-tetrahydroxy-10-(hydroxymethyl)-13-methyl-3-[(2R,3R,4R,5R,6S)-3,4,5-trihydroxy-6-methyloxan-2-yl]oxy-2,3,4,6,7,8,9,11,12,15,16,17-dodecahydro-1H-cyclopenta[a]phenanthren-17-yl]-2H-furan-5-one	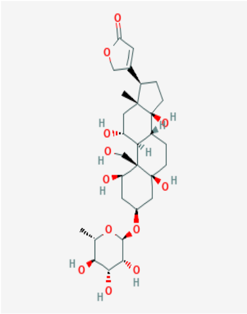	584.659 g/mol	-9.5
27.	6758	(2R,6aS,12aS)-1,2,6,6a,12,12a-hexahydro-2-isopropenyl-8,9-dimethoxychromeno[3,4-b]furo(2,3-h)chromen-6-one	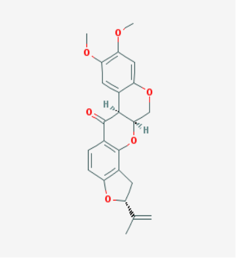	394.423 g/mol	-9.0
28.	5324346	2-[(E)-[5-methoxy-1-[4-(trifluoromethyl)phenyl]pentylidene]amino]oxyethanamine	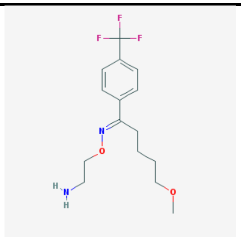	318.34 g/mol	-7.2

29.	124886	L-gamma-glutamyl-L-cysteinyl-glycine	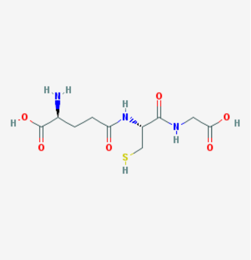	307.321 g/mol	-7.0
30.	6037	(2S)-2-[[4-[(2-amino-4-oxo-1H-pteridin-6-yl)methylamino]benzoyl]amino]pentanedioic acid	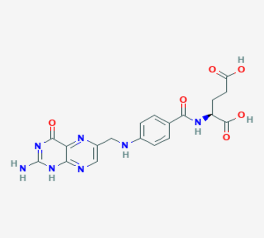	441.404 g/mol	-9.6
31.	1183	4-hydroxy-3-methoxybenzaldehyde	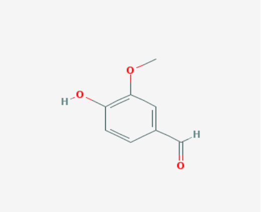	152.149 g/mol	-5.3
32.	171548	5-[(3aS,4S,6aR)-2-oxo-1,3,3a,4,6,6a-hexahydrothieno[3,4-d]imidazol-4-yl]pentanoic acid	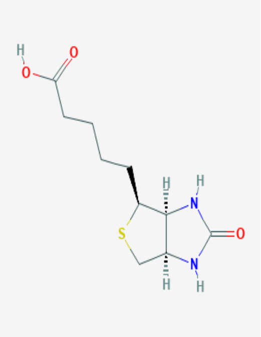	244.309 g/mol	-6.7

33.	6019	4,5-bis(hydroxymethyl)-2-methylpyridin-3-ol;hydrochloride	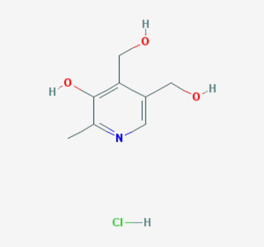	205.638 g/mol	-5.8
34.	7045767	(3R)-3-acetyloxy-4-(trimethylazaniumyl)butanoate	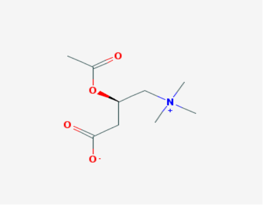	203.238 g/mol	-5.3
35.	9750	(2S)-2-amino-5-(carbamoylamino)pentanoic acid	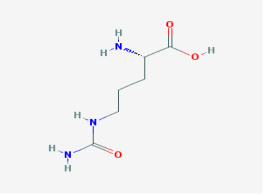	175.188 g/mol	-5.4
36.	10621	(2S)-5-hydroxy-2-(3-hydroxy-4-methoxyphenyl)-7-[(2S,3R,4S,5S,6R)-3,4,5-trihydroxy-6-[[(2R,3R,4R,5R,6S)-3,4,5-trihydroxy-6-methyloxan-2-yl]oxymethyl]oxan-2-yl]oxy-2,3-dihydrochromen-4-one	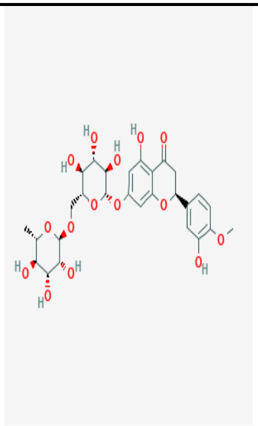	610.565 g/mol	**-11.4**

37.	637775	(E)-3-(4-hydroxy-3,5-dimethoxyphenyl)prop-2-enoic acid	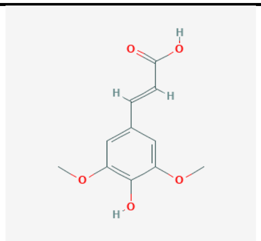	224.212 g/mol	-6.6
38.	2247	1-[(4-fluorophenyl)methyl]-N-[1-[2-(4-methoxyphenyl)ethyl]piperidin-4-yl]benzimidazol-2-amine	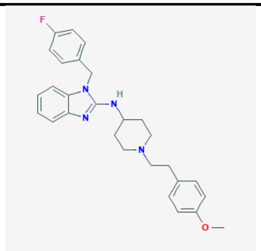	458.581 g/mol	-8.7
39.	1024	4,5-dioxo-1H-pyrrolo[2,3-f]quinoline-2,7,9-tricarboxylic acid	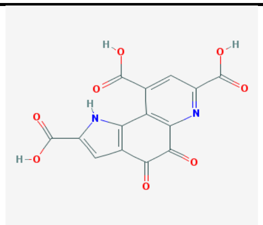	330.208 g/mol	-8.6
40.	673	2-(dimethylamino)acetic acid	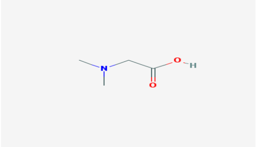	103.121 g/mol	-4.3

41.	5280804	2-(3,4-dihydroxyphenyl)-5,7-dihydroxy-3-[(2S,3R,4S,5S,6R)-3,4,5-trihydroxy-6-(hydroxymethyl)oxan-2-yl]oxychromen-4-one	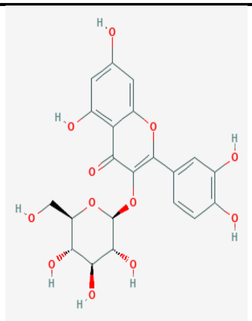	464.379 g/mol	-10.5
42.	5280795	(1S,3Z)-3-[(2E)-2-[(1R,3aS,7aR)-7a-methyl-1-[(2R)-6-methylheptan-2-yl]-2,3,3a,5,6,7-hexahydro-1H-inden-4-ylidene]ethylidene]-4-methylidenecyclohexan-1-ol	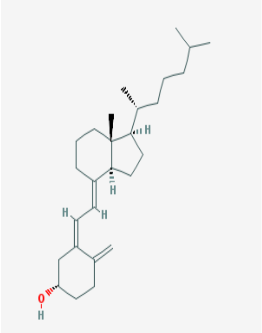	384.648 g/mol	-7.4
43.	5280483	2-methyl-3-[(E)-3,7,11,15-tetramethylhexadec-2-enyl]naphthalene-1,4-dione	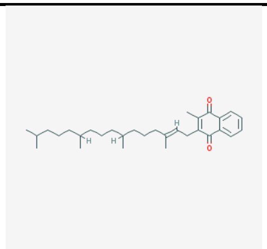	450.707 g/mol	-8.7
44.	11183027	2,3-dimethoxy-5-methylbenzene-1,4-diol	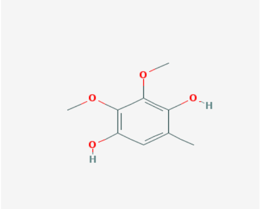	184.191 g/mol	-5.6

45.	689043	(E)-3-(3,4-dihydroxyphenyl)prop-2-enoic acid	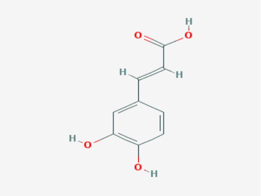	180.159 g/mol	-6.3
46.	9064	(2R,3S)-2-(3,4-dihydroxyphenyl)-3,4-dihydro-2H-chromene-3,5,7-triol	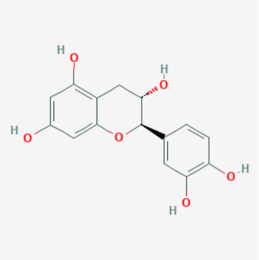	290.271 g/mol	-8.4
47.	442428	(2S)-7-[(2S,3R,4S,5S,6R)-4,5-dihydroxy-6-(hydroxymethyl)-3-[(2S,3R,4R,5R,6S)-3,4,5-trihydroxy-6-methyloxan-2-yl]oxyoxan-2-yl]oxy-5-hydroxy-2-(4-hydroxyphenyl)-2,3-dihydrochromen-4-one	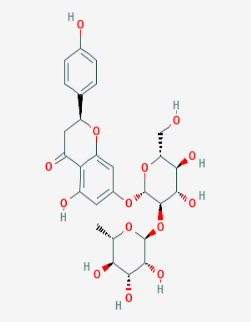	580.539 g/mol	-10.5

48.	83489	(2S)-2-(3,4-dihydroxyphenyl)-5-hydroxy-7-[(2S,3R,4S,5S,6R)-3,4,5-trihydroxy-6-[[(2R,3R,4R,5R,6S)-3,4,5-trihydroxy-6-methyloxan-2-yl]oxymethyl]oxan-2-yl]oxy-2,3-dihydrochromen-4-one	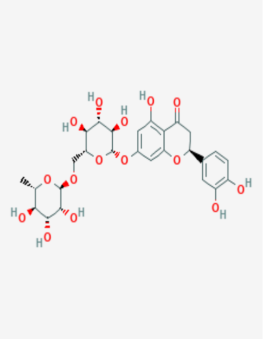	596.538 g/mol	-11.0
49.	11250133	(2R,3S)-2-(3,4-dihydroxyphenyl)-8-[(2R,3R,4R)-2-(3,4-dihydroxyphenyl)-3,5,7-trihydroxy-3,4-dihydro-2H-chromen-4-yl]-3,4-dihydro-2H-chromene-3,5,7-triol	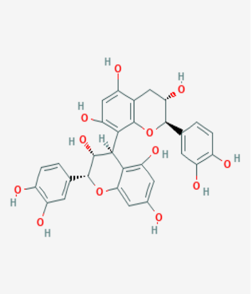	578.526 g/mol	-11.2
50.	1794427	(1S,3R,4R,5R)-3-[(E)-3-(3,4-dihydroxyphenyl)prop-2-enoyl]oxy-1,4,5-trihydroxycyclohexane-1-carboxylic acid	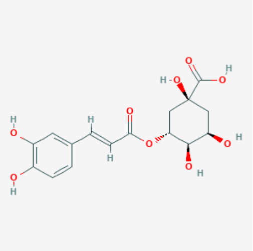	354.311 g/mol	-9.1

51.	164544	2-(4-hydroxy-3-methoxyphenyl)chromenylium-3,5,7-triol;chloride	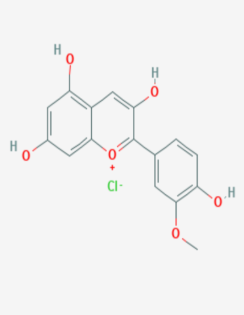	336.724 g/mol	-8.3
